# Electrical Characterization and Analysis of Single Cells and Related Applications

**DOI:** 10.3390/bios13100907

**Published:** 2023-09-26

**Authors:** Weitao Zhu, Jiaao Wang, Hongzhi Luo, Binwen Luo, Xue Li, Shan Liu, Chenzhong Li

**Affiliations:** 1 Clinical Medicine (Eight-Year Program), West China School of Medicine, Sichuan University, Chengdu 610044, China; justin_z020321@163.com (W.Z.); tttttrain_w@163.com (J.W.); 2 Department of Laboratory Medicine, The Third Affiliated Hospital of Zunyi Medical University (The First People’s Hospital of Zunyi), Zunyi 563002, China; hongzhiluo666@163.com; 3 School of Medicine, University of Electronic Science and Technology of China, Chengdu 610054, China; 202122130319@std.uestc.edu.cn; 4 Sichuan Hanyuan County People’s Hospital, Hanyuan 625300, China; liyixideyouxiang@163.com; 5 Sichuan Provincial Key Laboratory for Human Disease Gene Study, Department of Medical Genetics, Sichuan Academy of Medical Sciences & Sichuan Provincial People’s Hospital, University of Electronic Science and Technology of China, Chengdu 610072, China; 6 Biomedical Engineering, School of Medicine, The Chinese University of Hong Kong, Shenzhen 518172, China; chenzhongbiosensor@gmail.com

**Keywords:** single-cell analysis, electrical manipulation, single-cell application

## Abstract

Biological parameters extracted from electrical signals from various body parts have been used for many years to analyze the human body and its behavior. In addition, electrical signals from cancer cell lines, normal cells, and viruses, among others, have been widely used for the detection of various diseases. Single-cell parameters such as cell and cytoplasmic conductivity, relaxation frequency, and membrane capacitance are important. There are many techniques available to characterize biomaterials, such as nanotechnology, microstrip cavity resonance measurement, etc. This article reviews single-cell isolation and sorting techniques, such as the micropipette separation method, separation and sorting system (dual electrophoretic array system), DEPArray sorting system (dielectrophoretic array system), cell selector sorting system, and microfluidic and valve devices, and discusses their respective advantages and disadvantages. Furthermore, it summarizes common single-cell electrical manipulations, such as single-cell amperometry (SCA), electrical impedance sensing (EIS), impedance flow cytometry (IFC), cell-based electrical impedance (CEI), microelectromechanical systems (MEMS), and integrated microelectrode array (IMA). The article also enumerates the application and significance of single-cell electrochemical analysis from the perspectives of CTC liquid biopsy, recombinant adenovirus, tumor cells like lung cancer DTCs (LC-DTCs), and single-cell metabolomics analysis. The paper concludes with a discussion of the current limitations faced by single-cell analysis techniques along with future directions and potential application scenarios.

## 1. Introduction

Several decades ago, the introduction of the membrane-clamp technique revolutionized the study of cell physiology. It observes the function of individual ion channels in a variety of cells through a high-resolution electrochemical method. At the cellular level, the membrane-clamp technique allows the study of physiological processes, such as cellular signal transduction and synaptic transmission. These studies have contributed to a new understanding of the mechanism of certain disease processes and provided new ideas for the treatment of these diseases. Biological parameters extracted from electrical signals from the nervous system, muscles, heart, and other parts of the body have been used for years to analyze the human body and its behavior. In addition, electrical signals from cancer cell lines, normal cells, and viruses, among others, have been widely used for the detection of various diseases. Single-cell parameters such as cellular and cytoplasmic conductivity, relaxation frequency, and membrane capacitance are important. The physiological state of cells can change in response to stimulation, and therefore, the health state of cells can be characterized by measuring some specific cell markers [1]. Electrical parameters are original cell markers, which can identify diseases, enabling early diagnosis and prevention. The electrical properties of cells are often used to identify cells and describe their viability and growth, and these properties are closely related to cell structure and chemical composition [2]. Therefore, it is possible to obtain the dielectric properties of cells by quantitative analysis of their electrical parameters. The electrical properties of cells can provide key data that can provide insight into their complex physiological state. Electrical properties are important in cell quantification, isolation, and capture, and in single-cell characterization studies. For example, based on the deflection properties of a charged droplet in an electric field, the charging electrode charges the droplets, including the cells within, as the sample is ejected. The electrostatic properties of the cells determine the amount of charge they carry. They will be deflected from the mainstream in the electric field and move toward different electrode sheets, which achieves the purpose of sorting, separating, and capturing the cells [3].

Single-cell electrical manipulation can safely manipulate cells without causing harm, and analytical and numerical polarization models resulting from electric fields can be used to describe and characterize the dielectric electrophoretic behavior of cells [4]. Single-cell electrical manipulation enables the separation of individual cells to specific spatial regions for single-cell analysis, as well as the ability to isolate and characterize rare cells. In addition to the detection of membrane, cytoplasm, and nuclear antigens, single-cell analysis can also detect whole cells and cellular components, such as organelles, nuclei, DNA, RNA, chromosomes, cytokines, hormones, and protein content, which can also be studied by single-cell manipulation. Single-cell analysis also enables further exploration of cell proliferation and cell cycles [3].

There are many techniques that can be used to characterize biomaterials, such as nanotechnology, microstrip cavity resonance measurement, etc. Cellular techniques can be divided into single-cell analysis and cell identification. Single-cell analysis uses patch clamps and nanoprobes, and the latest technologies include microfluidic and micro-electrical impedance spectroscopy.

Currently, most of the articles in the literature only introduce single-cell related techniques in different fields, and there is no systematic overview of the various operations and applications of single-cell analysis. In this review, we review the methods for single-cell sorting and isolation and discussed their advantages, disadvantages, and possible application scenarios. This article also reviews single-cell operations, summarizes several methods for extracting electrical parameters, and introduces their structural characteristics and application fields. Next, this article illustrates the specific analysis and applications of single-cell sequencing. Finally, we summarize the current shortcomings and problems of single-cell sequencing, provide solutions, and tentatively give future application directions and prospects.

## 2. Single-Cell Sorting and Separation

Generally, biological samples used for testing are often a mixture of numerous normal and unimportant cells, with only a small proportion of cells of real research and reference value. Overall analysis of biological samples provides only average information on mixed-cell populations, while a small proportion of significant cell subpopulation information is lost in the background. For example, in high-throughput sequencing analyses of tumor tissue, mixed samples of millions of cells are typically analyzed simultaneously. This method of analysis reflects the overall genomic characteristics of the cells, but may ignore the heterogeneity of tumor cells and lead to dilution of the genetic material of cells of low abundance but important discriminatory significance, such as circulating tumor cells (CTCs) and cancer stem cells (CSCs), making them difficult to distinguish [5]. The single-cell sorting and separation technique allows for the precise selection of certain cells of greater value from a mixed population of multiple cell types and amounts, greatly facilitating the subsequent single-cell analysis for these cells. The selection of the sorting method depends to a large extent on the sample to be separated and the subsequent analytical operations to be performed. In review, single-cell separation and sorting are quite different techniques. Single-cell sorting techniques focus on separating specific cell populations from mixed samples containing multiple cell populations based on their different biological or physical properties. Single-cell separation techniques, on the other hand, concentrate on separating individual cells from large pieces of tissue or aggregated cell samples. Here we discuss a few typically related techniques. Micropipette isolation is a common method for single-cell separation. The separation and sorting system, the cell selector sorting system, and the DEPArray sorting system can all satisfy the need of both single-cell separation and sorting. Microfluidics are a popular single-cell sorting approach.

Micropipette isolation is an earlier technique for single-cell isolation, which is conducted under a high-power microscope using micromechanical manipulators or visual tweezers [6]. For example, in single-cell studies of spermatozoa, researchers manipulated sperm samples under a microscope with a manual Cell Tram Oil microinjector (Eppendorf), manually isolated sperm cells using a sterile glass micropipette with an inner diameter of 20 mm (Eppendorf Transfer Tip [ES]), and placed them on glass slides fitted with adhesive. Sperm cells were manually isolated and placed on glass slides with adhesive press-to-seal silicon isolation wells (Invitrogen). The cells were then also rinsed through a rinse well containing cold PBS-BSA (50 mL), and each cell was drawn into and out of the microcapillary approximately 10 times before final capture [6]. This method is low-cost, accurate and can effectively control the selection, transfer, and release of target cells, but it is time-consuming, has low throughput, and easily causes mechanical damage to target cells. This method is suitable for isolating a small number of target cells from the whole cell population [7].

As the study of living cells is more valuable, it is important to maintain the physiological activity of the cells in single-cell analysis. As the application of single-cell isolation techniques has expanded further, the use of micropipette isolation methods, which can cause damage to cells, has declined, and techniques that can be performed non-destructively are becoming more widely used. The separation and sorting system (dual electrophoretic array system) is a semi-automatic sorting system that separates rare cells from the mixed-cell population by fluorescent labeling and places the sorted and captured target cells in appropriate locations for subsequent sequencing analysis [8]. The DEPArray sorting system (dielectrophoretic array system) is also a semiautomatic sorting system which separates rare cells from a mixed-cell sample. Single cells are captured by the microelectrodes arranged on the chip. Then the microelectrodes are controlled to move the target cells to a certain position on the chip and transfer them to suitable media to complete the following sequencing analysis. The cell selector sorting system automatically separates rare cells from mixed-cell populations using a multifunctional robotic system. It automatically searches and realizes single-cell sorting, and mechanically separates target cells or clones directly without affecting cell vitality. It can conduct cell sorting by observing cell images in real time with high precision [9]. However, the technologies mentioned have shared disadvantages: they are time-consuming and require a small sample. Therefore, the samples always need to be divided and enriched before they can be input to these systems [10].

Microfluidic technologies provide alternative pathways in single-cell analysis with a larger sample scale and higher efficiency. Microfluidics can complete single-cell sorting, cleavage, and amplification, and have the characteristics of high throughput, small reaction volume, less pollution, and little influence on sequencing. However, the disadvantages of high cost and low capture rate for viscous and non-spherical cells are unmissable [11]. Among the microfluidic technologies, the droplet microfluidic method is gaining increasing attention and is widely used in many different circumstances. This approach uses many droplets formed by reagents in a water-in-oil emulsion. Every individual mixed droplet is an independent reaction unit; different droplets are separated and connected by a continuous flow of inert oil. Therefore, the chemical reactions of different droplets are isolated. There is no interference among droplets [12]. The advantage of this technology is that single-cell operations including lysis, separation, and extraction can be easily performed in droplets. Such operations also have a high throughput. However, there are currently some bottlenecks in droplet microfluidics, as processes such as washing and buffer exchange are still difficult to implement on a large scale [13].

In addition, valve devices are also commonly used in microfluidic technologies. The valves connect microfluidic chambers, which play an important role in flow control and cell confinement [14]. These valves make it easy to add and remove reagents and capture, mix, split, and lyse analytes to be analyzed in different reaction chambers for a range of different analyses and manipulations. Furthermore, as such operations are performed at the single-cell level, each cell can be precisely selected for analysis. In addition, the opening and closing of the valves can be controlled automatically by a computer program, thus greatly increasing the automation scale of the analysis work and saving time and labor costs [15].

## 3. Single-Cell Manipulation

After a specific single cell is isolated, it needs to be manipulated by a specific method to obtain the interesting biological parameters. At the single-cell level, many cell characteristics are of interest to study [16]. For instance, there are no two cells from a shared genetic group that are totally the same. Such differences are expressed as phenotype heterogeneity at the single-cell level, which is essential for accurate explaining of diagnostic and treatment results of diseases [17]. The heterogeneity among genetically identical cells also plays an important role in the analysis of cancer metastasis [18], drug resistance [19], and stem-cell differentiation [20]. Methods for implementing electrical impedance measurements in microfluidic devices generally include single-cell amperometry (SCA), electrical impedance sensing (EIS), impedance flow cytometry (IFC), cell-based electrical impedance (CEI), microelectromechanical systems (MEMS), and integrated microelectrode array (IMA).

A common goal of single-cell analysis technology is to try to achieve high throughput and label-free analysis without damaging the cells. In general, fluorescent labelling techniques require many sample preparation steps and the removal of the labelled dye is always indispensable before subsequent processing can take place, whereas label-free methods eliminate these steps. However, it is important to note that although label-free cell isolation methods based on intrinsic characteristics such as cell–cell morphology, size, and deformability can provide information on cell phenotypes, these methods are inferior in specificity and sensitivity to the fluorescent-antibody-labelled methods used in conventional stream cytometry [21]. Therefore, although classic technologies such as patch-clamp and fluorescent probes are quite effective tools, some new approaches such as SCA, EIS, and IMA are in some ways superior.

First of all, single-cell ammeter is an intracellular nano-electrochemical sensor. Benefiting from the advantages of the tiny size and high spatial resolution of the nano-electrode, SCA can provide delicate electrochemical analysis of the internal environment of a single cell [22]. When analyzing a single cell, a nanoelectrode is usually placed into the cell using a robotic hand [23,24], while the position of the electrode tip is observed by scanning electrochemical microscopy (SECM) [25]. Nanoelectrodes are typically placed in the cell membrane [26], near mitochondria [27], or in the nucleus [26] for electrochemical measurements. CA is an invasive single-cell manipulation method, and it is important to monitor cell leakage. Researchers have typically used the single electron mediator Ru (NH3)63+, which cannot cross the cell membrane, to monitor leakage. Ru (NH3)63+ is added to the extracellular media, and if this medium cannot be detected intracellularly by amperometry, then the cell to be tested is not leaking [27].

SCA is interesting for the study of substance metabolism within single cells. For example, a researcher has used platinized carbon nanoelectrodes to quantify intracellular ROS and RNS, by which it was demonstrated that the levels of ROS and RNS in cancerous MDA-MB-468 and MDA-MB-231 cells were much higher than those in non-cancerous MCF-10A cells [28]. Using the same method to detect intracellular ROS and RNS levels, researchers also successfully explored the effects of anticancer drugs on the activity of PC-3 and 22RV1 cells [29].

In addition, many common biometabolites can be quantitatively measured by SCA, such as H_2_O_2_ [30] and NADH [31]. Based on the principle that Prussian blue can reduce hydrogen peroxide, researchers have quantitatively measured H_2_O_2_ in MCF10A, MCF7, and MCF7/HER2 cells [32] and murine macrophages [30] using carbon nanoelectrodes containing Prussian blue. Some researchers have quantitatively detected intracellular NADH in MCF-7 cells using asymmetric nanopore electrodes. This electrode is coated with a layer of gold on the inside of the nanopore and functionalized with the addition of catecholamines [31].

Moreover, to study the insulin release process, researchers placed an ammeter at the tip of the nanoelectrode, consisting of intracellular vesicle impact electrochemical cytometry (IVIEC), to quantify the process of secretory vesicle production by β-cells. Since insulin is more difficult to detect electrochemically and 5-HT, which is in the same secretory vesicle as insulin, is easily oxidized and detected by the electrode, a disk carbon fiber electrode (CFE) was used to quantify the amount of 5-HT released from a single secretory vesicle. Pancreatic β-cells can be induced to produce secretory vesicles and exocytosis them by changing the concentration of external ions. By quantifying the amount of material released from each vesicle at a time, researchers demonstrated that the release of chemicals from β-cells is partial, with only about one-third of the release occurring at a time (39,317 ± 1611 serotonin molecules within a single nanoscale vesicle and 13,310 ± 1127 serotonin molecules released from a single cell per stimulation of exocytosis) [33].

Overall, the single-cell ammeter is very widely used for electrochemical detection of various intracellular substances and is a very effective approach. However, it requires the electrodes to be pierced into the cells, a process with limited success, which can also cause some damage to the cells. Therefore, it has certain limitations in its use and requires strict detection of cell leakage.

**Figure 1:**
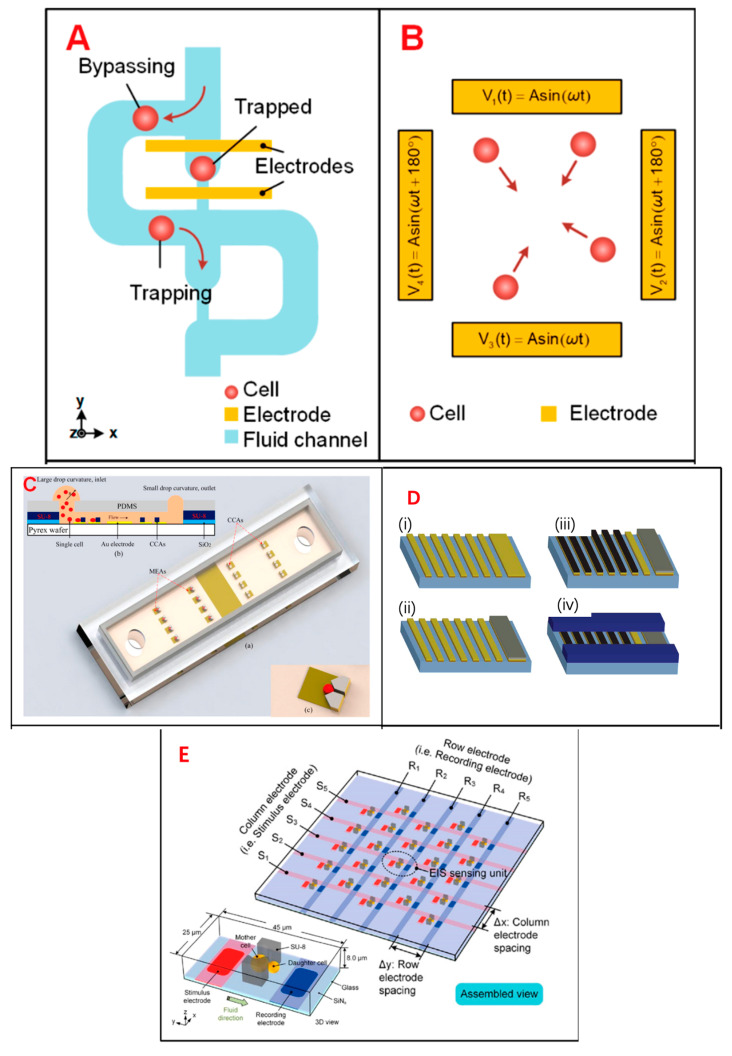

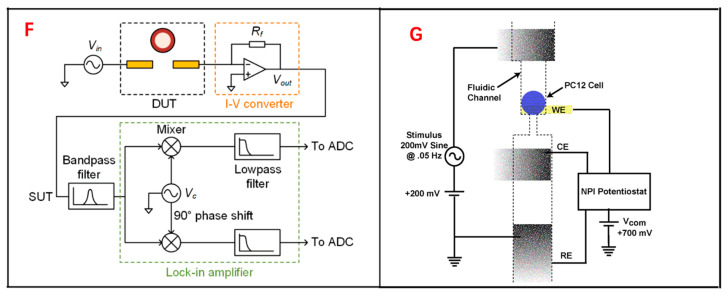
Different designs of EIS devices: schematics of a single-cell EIS sensing system, a multilayer microelectromechanical system (MEMS) device for single-cell electric impedance spectroscopy and electrochemical analysis, and an IFC device used for tumor analysis. (**A**) Schematics of μ-fluidic traps, which can immobilize single cells within a cellular EIS sensing device. Reprinted from Ref. [34]. (**B**) A quadrupole electric device to gather cells towards the center. Reprinted from Ref. [34]. (**C**) A 3D single-cell culturing device, which can reveal HeLa cell migration. (a) 3D model diagram of the device (b) Schematic diagram of the operating principle of the device (c) Schematic diagram of the single cell capture device. Reprinted with permission from Ref. [56]. Copyright 2013 American Chemical Society. (**D**) The production process of MEMS. (i) The first layer: deposit and arrange the Ti/Au layer. (ii) The second layer: sputter deposit the Ag layer and chloride. (iii) Electroplate platinum black to select electrodes. (iv) Spin SU-8 2010 to a ~12-μm thickness and subsequently arrange it. Reprinted with permission from Ref. [57]. Copyright 2008 IEEE. (**E**) A microelectrode array device to fix a single cell for EIS measurement. Reprinted with permission from Ref. [58]. Copyright 2021 John Wiley and Sons. (**F**) Schematics of a single-cell impedance sensing system with lock-in amplifier. Reprinted from Ref. (**G**) A circuit model to analyze the release of neurotransmitters from a single PC12 cell during electrical stimulation. Reprinted with permission from Ref. [58]. Copyright 2021 John Wiley and Sons.

EIS is a powerful tool that allows rapid, noninvasive, and label-free access to the electrical parameters of single cells. The electrical parameters of single cells, including equivalent cell resistance, membrane capacitance, and cytoplasmic conductivity, are closely related to the biophysical properties and dynamic activities of cells such as size, morphology, membrane integrity, growth state, and proliferation [34]. With miniaturization, low cost, geometric size comparable to cell size, and flexible structure design, microfluidic technology is a powerful tool for single-cell analysis, providing operation and analysis methods at the single-cell level (Figure 1) [35]. EIS sensing is used to select the most sensitive frequency for subsequent high-speed analysis or long-term monitoring of cell behaviors and phenotypes. The measured EIS can characterize various cellular physiological processes such as adhesion, growth, division, differentiation, proliferation, and cellular structure formation. The electrical impedance properties of single cells can reveal the complex physiological state of cells [36]. Single-cell electrical-impedance-based biosensors can detect a variety of biological parameters and do not rely on fluorescent labeling. At lower frequencies (100 KHZ to 1 MHz), electrical impedance refers to the size information of the cell, while at moderate frequencies (about a few MHz), electrical impedance refers to the membrane capacitance of the cell, and at higher frequencies, electrical impedance refers to the conductance of the intracellular organelles of the cell [37]. To accurately classify different cell subsets, it is necessary to use a combination of multi-frequency impedance signals and a variety of biophysical parameters to enrich the characteristic information of different cells, which is conducive to improving the phenotypic resolution [38]. Electrical impedance integrated microfluidic devices have been widely used for cell-based single-cell analysis.

Integrated microelectrode array (IMA) biosensors could also be explored for single-cell analysis. The micro-processing and single-cell level operation of IMA chips are realized by the surface chemical modification of IMA chips. Important sensing parameters are identified, including specific cell membrane capacity, cell membrane resistivity, and cell-substrate average separation.

Analysis of the frequency-dependent properties of the single-cell covered microelectrode impedance and the IMA sensor circuit response revealed a frequency band in which the electrical properties of single cells can be determined for cellular biosensing applications. For instance, a single fibroblast cell (NIH3T3) could be fixed on peptide microelectrodes, such as lysine–arginine–glycine–aspartic acid (KRGD) short-peptide-modified or fibronectin extracellular cell-adhesion-molecule-modified microelectrodes. Therefore, under certain frequency band of 1 to 10 kHz, the impedance value changes of a cell–electrode heterostructure can be measured [32].

**Figure 2:**
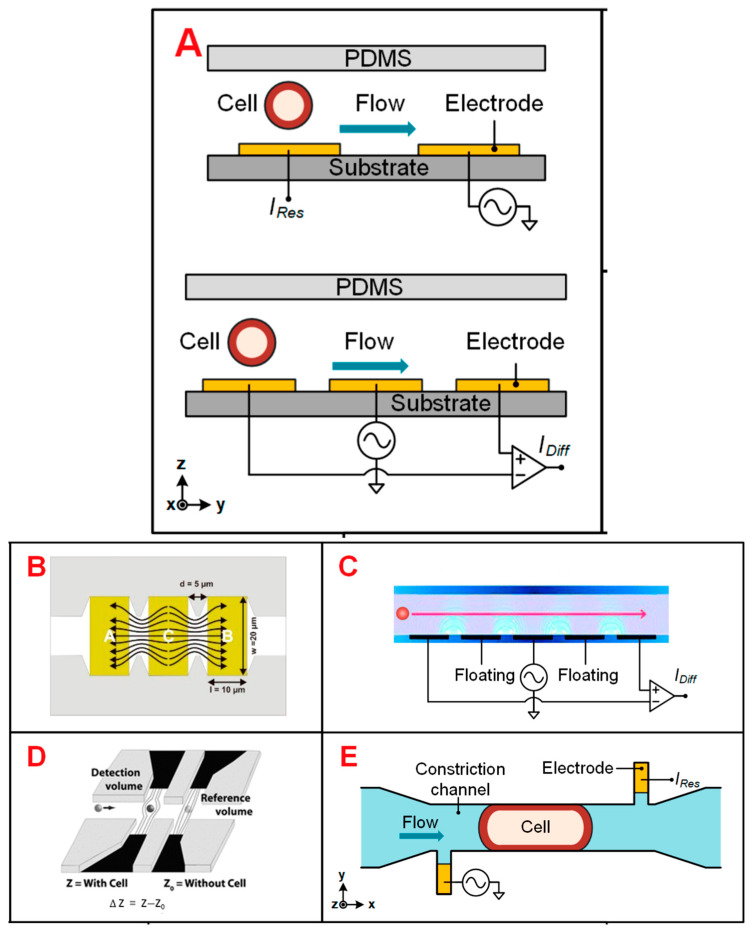
Different IFC devices’ designs. (**A**) Schematics of coplanar electrode configurations for absolute and differential measurement. Reprinted from Ref. [34]. (**B**) The coplanar electrode, which has a larger electrode area exposed to the medium. Reprinted from Ref. [61]. (**C**) Coplanar electrodes with two extra floating electrodes. Reprinted from Ref. [62]. (**D**) Electrodes of liquid material. Reprinted with permission from Ref. [63]. Copyright 2010 Royal Society of Chemistry. (**E**) Asymmetrical liquid electrodes, with the constriction channel through which cells flow. Reprinted from Ref. [34].

IFC is a high-throughput single-cell analysis method with miniaturization, low peripheral requirements, and flexible integration of query units, which has been widely used in many fields like Table 1 [39]. IFC measures the change of the response current caused by a single cell passing through the schema-shaped electrode in the microfluidic channel (Figure 2). The distribution of the AC electric field in the channel determines the sensitivity of the IFC device, so the electrode configuration should be carefully considered [40].

**Table 1:** An aggregation of the recent advances in cell impedance research by the IFC method.

Category	First Author (Year)	Target Cells	Application	Ref.
**Tumor cells**	Desai (2019)	Thyroid, Breast, Lung, and Ovarian cancer cells	Cell recognition	[41]
	Ren (2019)	MDA-MB-231 cells	Cell recognition	[42]
	McGrath (2020)	Six types of pancreatic ductal	Cell screening	[43]
	Ostermann (2020)	Adenocarcinoma cell U937 cells	Viability assay	[44]
	Zhang (2020)	A549 and Hep G2 cells	Cell screening	[45]
**Plant cells**	Impe (2019)	Wheat pollen Hazelnut pollen	Viability assay	[46]
	Ascari (2020)	Wheat microspore	Viability assay	[47]
	Canonge (2020)	Herbaceous	Monitoring androgenesis process	[48]
	Han (2020)	*Arabidopsis thaliana* and woody *Populus trichocarpa*	Cell screening	[49]
**Microbes**	Xie (2019)	*S. cerevisiae*	performance assessment	[50]
	Opitz (2019)	*S. cerevisiae*	Viability assay	[51]
	Bertelsen (2020)	*E. coli*	Determination of the viability of *E. coli*	[52]
	Spencer (2020)	*K. pneumoniae*	Antimicrobial susceptibility tests	[53]
**Stem cells**	Song (2016)	Mesenchymal stem cells	Monitoring differentiation process	[54]
	Xavier (2017)	Skeletal stem cells	Monitoring differentiation process	[55]

In single-cell studies targeting biological processes, the real-time nature of the measurement results is of great importance. Although EIS, IFC, and other methods are label-free, high-throughput and unharmful to cells, their measurement results are not as good as the CEI method in real time analysis. CEI biosensors have been extensively explored, and the technique does not require cellular manipulation and provides real-time dynamic measurements of receptor-mediated cellular changes. An electro-biosensor measures the impedance of a cell grown on a surface embedded with an electrode when the cell is exposed to an electric field generated by a continuous sweep of an AC voltage over a range of frequencies. Impedance depends on the number, size, and shape of cells on the electrode surface, the distance between cells and the electrode surface, and cell–cell contacts [59]. CEI assays provide more comprehensive efficacy predictions than many other assays since both activating signaling events and down-regulation events contribute to the overall CEI response. Meanwhile, due to the comprehensive nature of the detected response, CEI can also detect biased compounds and monitor the cellular toxicity of compounds in real-time [60]. Screening with CEI has less bias because the readings are not concentrated into one pathway. CEI assays can be used for initial screening of known targets, especially smaller compound libraries, and for understudied targets where downstream signaling pathways are still unclear [59].

On the other hand, microfluidic technology also has diverse applications in single-cell manipulation. In recent years, researchers have placed increasing emphasis on the efficiency of single-cell manipulation. A higher degree of automation is therefore becoming increasingly important for single-cell manipulation techniques. MEMS is one typical example. MEMS technology, in particular, enables the study of individual cells through simplified semi-automated high-throughput methods. With the rapid development of MEMS technology, a number of micro-scale devices have already been constructed for bioanalysis at the single-cell scale. In some cases, MEMS methods have inherent scaling advantages [64] and have been used to study various cell types, including red blood cells [65], lymphocytes, and others.

## 4. Single-Cell Analysis and Application

**Figure 3:**
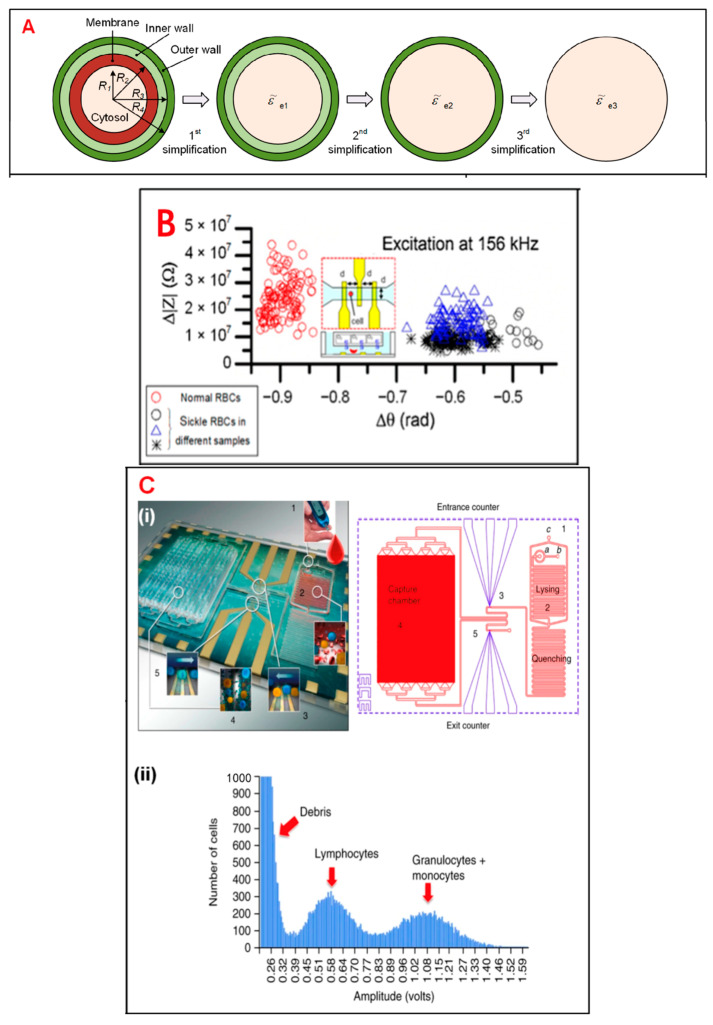

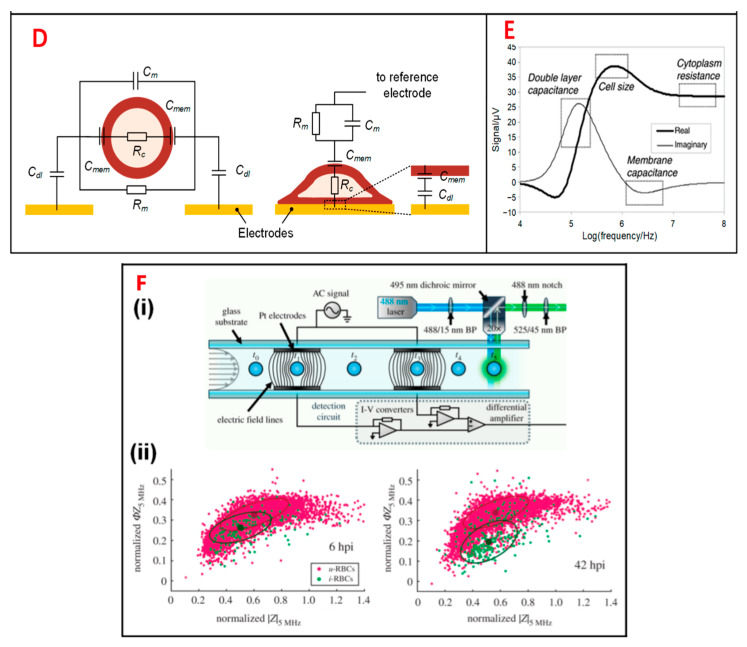
Electrical impedance sensing technique for single-cell analysis. (**A**) Electrical model and equivalent circuit models (ECMs) of a single cell. The mode is simplified to a homogeneous sphere. Reprinted from Ref. [34]. (**B**) Measurement of electrical impedance for normal and sickle RBCs at 156 kHz. Reprinted with permission from Ref. [66]. Copyright 2018 Elsevier. (**C**) (**i**) Pictures of immunocapture biochip. (**ii**) Pulse amplitudes of impedance signals which show the distribution of cells by size. Reprinted with permission from Ref. [67]. Copyright 2016 Nature Publishing Group. (**D**) The cell mode is suspended between a pair of sensors and adhered on a sensor. Reprinted from Ref. [34]. (**E**) Simulation results of an ECM model, which show different frequency domains related to cell parameters. Reprinted with permission from Ref. [68]. Copyright 2001 Royal Society of Chemistry. (**F**) (i) Schematic of an IFC device. (ii) Measurement of impedance of infected and uninfected RBCs after 6 and 42 h. Reprinted from Ref. [69].

In general, living cells are usually of greater research interest due to their more significant biological properties and applications. Therefore, whether analysis can be performed while maintaining the activity of the target cells is a hot issue in single-cell technology. Most single-cell analysis techniques have the advantage of not damaging the cell cytosol. For example, single-cell sequencing is a non-invasive method for tumor diagnosis and prognosis prediction. Single-cell sequencing can obtain comprehensive information, assist in early diagnosis of tumors, select the best therapeutic drugs, and monitor recurrence. The integrated analysis of single-cell sequencing with other omics can also provide valuable information that can promote the development of precision medicine in cancer. In addition, some researchers have combined on-chip oxygen control with a single IFC chip for sickle cell disease diagnosis and monitoring, and the living single-cell-based analysis results proved to be accurate and detailed (Figure 3) [66].

Single-cell technology can also be used in biological tissue biopsies. Circulating tumor cells (CTCs) are tumor cells that originate from epithelial primary or metastatic tumors and enter the bloodstream with high viability and potential for metastasis. Liquid biopsy of CTCs can monitor tumor progression in real time [70]. Tumor metastasis is one of the main causes of death in cancer patients. CTCs can provide early warning of tumor heterogeneity and drug resistance and identify the mechanism of tumor occurrence and development [71]. Current methods for detecting tumor metastasis are mainly based on imaging, but early tumor metastasis is difficult to detect at the cellular level, even when viewed under high-resolution images. However, through high-resolution imaging combined with dynamic monitoring of the number and nature of CTCs, potential clues of tumor lesion metastasis can be found, providing potential for early targeted therapy [5]. Often, high-throughput sequencing analyses of tumor tissues ignore their heterogeneity because they target mixed samples of millions of cells that only reflect the overall genomic characteristics of the cells, while ignoring genetic material from low-abundance but functionally important cells, such as CTCs and cancer stem cells [72]. The separation of CTCs can be divided into two steps: enrichment and capture. The technology is mainly based on cell surface labeling and microfluidic chips. Enrichment based on cell surface markers generally includes positive and negative selection. This method utilizes antibodies, such as anti-epithelial cell adhesion molecules (EpCAM) and cytokeratin (CK), to capture and enrich tumor cells from the epithelium, while leucocyte-derived antibodies are utilized to eliminate white blood cells [73]. Based on the enrichment of microfluidic chips, peripheral blood mononuclear cells (PBMCs) of tumor patients were first isolated according to the biological and physical characteristics of tumor cells. The PBMCs were then slowly passed through a microfluidic chip coated with EpCAM antibodies under slow laminar flow control. EpCAM+ cells were captured and bound to the bottom of the chip, while other lymphocytes flowed out with the fluid [21].

In fact, due to the small amount of PBMCs in peripheral blood, PBMCs can only reflect the occurrence of disease to a certain extent. In contrast, whole genome RNA expression profiling can reflect the occurrence of disease more comprehensively. Some researchers have taken bacterial meningitis as an example. The researchers extracted whole blood RNA from a group of patients for gene expression profiling and confirmed the expression levels of 10 functionally related genes (CD177, IL1R2, IL18R1, IL18RAP, OLFM4, TLR5, CPA3, FCER1A, IL5RA, and IL7R) with a high degree of statistical significance by real-time quantitative PCR (qRT). The researchers ultimately demonstrated that whole blood RNA analysis could better reflect the occurrence and course of the disease, proving that the transcript levels of certain immune genes are associated with pathogens with some specificity [73]. The authors also suggested that whole blood RNA analysis could also reveal some specific cellular activations [73]. Combining whole blood RNA analysis with single-cell gene analysis can better assist the diagnosis and treatment of diseases, which is a promising direction.

**Figure 4:**
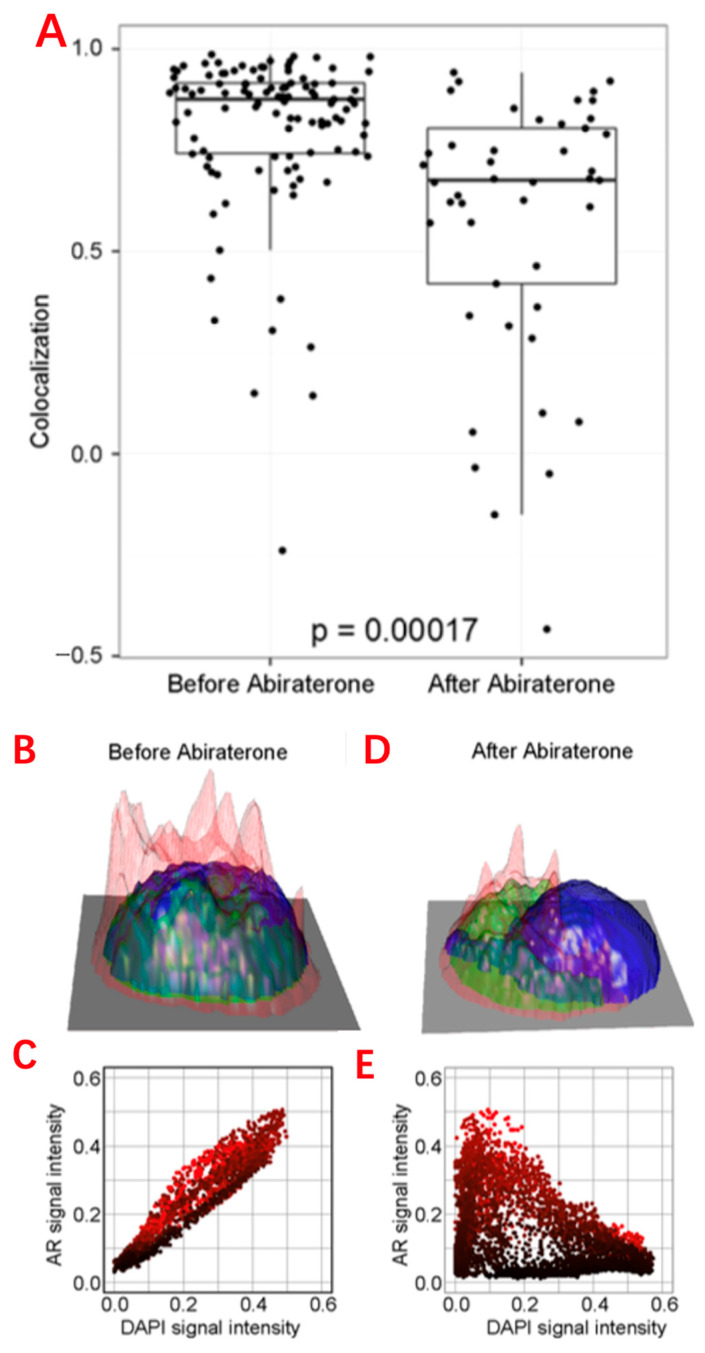

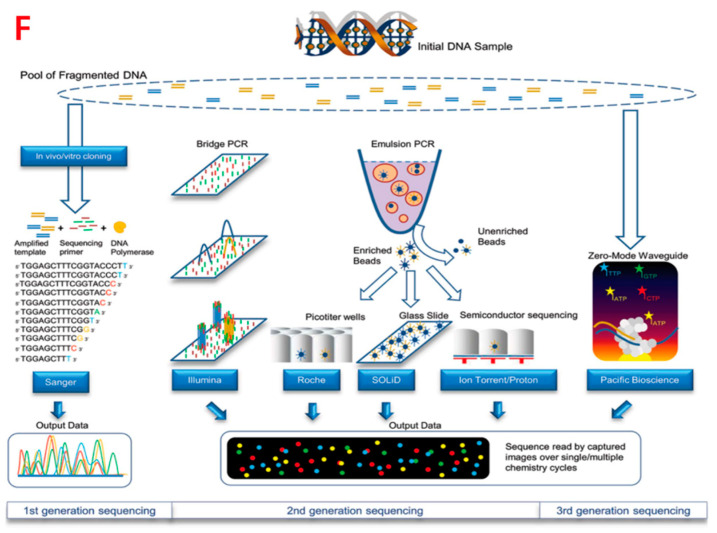
Single-cell analysis applications in CTCs. (**A**) Comparison of the AR subcellular localization in each CTC identified in the blood before and after 9 weeks of abiraterone treatment, which reveals the intracellular colocalization relationship between the AR and DAPI signals of CTCs. Reprinted from Ref. [5]. (**B**,**D**) Height maps of AR signal intensity of a single CTC, before and after 9 weeks of treatment. Reprinted from Ref. [88]. (**C**,**E**) AR versus DAPI signal intensities for each pixel inside the single CTC, revealing the relationship of nuclear exclusion as a negative correlation and nuclear localization as a positive correlation. Reprinted from Ref. [88]. (**F**) Different sequencing approaches. Technical features include sample preparation, sequencing chemistries, and data output formats. Reprinted with permission from Ref. [89]. Copyright 2014 SAGE Publications.

CTCs are mainly used in the following aspects: to reduce the interference of tumor heterogeneity, and to compare the differences between single-cell genomes, transcriptomes, and epigenomes between the tumor primary site and metastatic site and between peripheral blood CTCs and metastatic lymph nodes [74]. Single-cell transcriptome sequencing analysis of CTCs can be used to understand the therapeutic effect of tumors, as well as the genetic heterogeneity, evolution, and drug resistance of tumor cells (Figure 4). Surface markers and cell sizes of CTCs vary from tumor to tumor. Most CTCs are aggregated, which is also a significant feature of tumor stem cells, and the presence of CTCs in the peripheral blood of tumor patients can indicate the progression of the tumor [75]. For rare peripheral blood CTC detection, traditional single-cell sorting methods, such as fluorescence activated cell sorting (FACS), are no longer suitable. Instead, micromanipulation, microfluidic technology, and cell selection systems are used.

Single-cell technology can also be combined with viral recombinant technology for the precise treatment of human diseases. Studies have demonstrated gene therapy at the single-cell level, presented experimental and computational methods for parallel characterization of recombinant adeno-associated virus (rAAVs) tropism, and enabled safe and precise gene delivery vectors. Recombinant AAVs (rAAVs) are the gene delivery vector of choice for many studies because of their broad viral tropism, ability to transect dividing and nondividing cells, and their ability to ensure long-term transgene expression with stability and persistence [76]. However, rAAV has poor target specificity and a relatively low therapeutic index for systemic gene therapy [77], and optimized AAV gene delivery vectors that can be used for cell-type-specific delivery are urgently needed [78]. Single-cell RNA sequencing (scRNA-seq) enables comprehensive analysis of the transcriptome at the entire-cell-type level. High-throughput single-cell transcriptome analysis can aid in further understanding AVV. Studies provided tropism information beyond several AAV variants and beyond the predominant cell types.

Additionally, the use of high-throughput RNA Seq on samples with the rRNA removed allows for the detection of almost all coding and non-coding RNA species in a given sample [79]. Such studies provide a large amount of data to reference for single-cell RNA sequencing analysis. Combining the two and carrying out single-cell sequencing analysis based on sequencing of mass samples is also a promising research direction.

Outside of disease research, single-cell sequencing technology can also be used to reveal the development process of various human tissues. For example, single-cell sequencing has been applied to the research of cortical cells of the nervous system, which assists the analysis of single cells in the developing human prefrontal cortex from 8 to 26 weeks of gestation, identifying cell types and subtypes within major categories [80].

The IFC device mentioned above is one of the most widely used single-cell analysis techniques. IFC has been used to classify two tumor cell lines, A549 and H1299 (human lung alveolar-like cell line, commonly used in non-small-cell lung cancer respiratory research), according to differences in cell membrane capacitance and cytoplasmic conductivity [81]. Other researchers have used IFC to isolate lung cancer DTCs (LC-DTCs) from red blood cells, peripheral blood mononuclear cells (PBMCs), and normal lung cells based on impedance amplitude [41]. Another investigator developed an IFC device to isolate individual PDAC tumor cells against a xenograft [43]. They found that the phases of impedance signals in six PDAC cells were correlated with specific gene expressions. The combination of individual intrinsic bioelectrical markers, such as membrane capacitance, cytoplasmic conductivity, and cell diameter, can significantly improve the classification accuracy of both A459 and HEPG2 tumor cells. In addition, some researchers used a commercial IFC device and found that necrotic and surviving U937 human lymphoma cells could be clearly distinguished based on the phase of the impedance signal [44]. In addition to its application to research directly related to humans, IFC equipment has been widely used for the detection, isolation, and activity analysis of unicellular microorganisms. Studies have demonstrated that IFC devices can detect bacteria based on cell size [82], can accurately measure the diameter of different bacteria [77], and can achieve higher sensitivity and detection throughput of bacterial size [83].

On the other hand, single-cell analysis techniques are of interest in the field of metabolism and help to gain insight into the relationship between the physiological behavior of single cells and their chemical components. As the ultimate products of cellular biochemical reactions, metabolites can accurately and in a real-time manner reflect the biochemical phenotypes of cells. Moreover, metabolite features can be converted into signals, such as electrochemical, optical, or mass-to-charge ratio. They can be measured relatively easily by electrochemical, optical, and mass spectrometry detection methods [84]. Therefore, they are generally regarded as the research objects of cellular metabolic processes. As a result of the heterogeneity of cells and the extremely fast turnover rate of some metabolites, traditional methods for studying the state of a large number of cells struggle to accurately reflect the metabolic processes of individual cells [85]. The introduction of single-cell analysis techniques has solved this problem well. Based on the traditional nanoelectrospray ionization mass spectrometry (nano-ESI-MS) technique, some researchers have further designed induced nano-ESI-MS. The new technique, which uses high-voltage alternating current to generate an inductive electrospray charge, targets the metabolite molecules, then measures them by the current, resistance, and voltage changes in the electrodes. It was used to study the metabolism of the internal metabolites of single neurons and revealed a novel pathway for brain glutamate biosynthesis [86]. Another technique, pulsed direct-current electrospray ionization mass spectrometry (pulsed-dc-ESI-MS), is often combined with microwell-based droplet microextraction and has been applied to the study of phospholipids and glucose phosphates in single cells [87]. However, several of the MS methods mentioned above use manual capillary probes for sampling, which makes their analytical efficiency more limited. To solve this problem, researchers developed a novel asymmetric serpentine channel microfluidic chip and combined it with pulsed electric-field-induced electrospray ionization high-resolution mass spectrometry (chip-PEF-ESI-HRMS). This method suspends cells in physiological saline droplets and uses a microfluidic device for continuous cell separation and inertial focusing operations. In this method, the analysis is performed in a near-physiological environment, and the efficiency is up to 80 cells per minute, it can be stable for more than 3 h, and 3000 cells can be analyzed consecutively in a single experiment. The researchers used this method to label 120 cancer cell metabolites and successfully distinguished two different cancer cells (MCF7 and HepG2 cells) based on single-cell metabolic profiles. This method can efficiently obtain metabolomic information of various single cells under near-physiological conditions, which has wide application prospects [85].

## 5. Conclusions

In this review, we have summarized the methods for single-cell sorting and isolation, and specific cell sorting procedures were introduced. This article next reviewed single-cell operations, including impedance flow cytometry, electrical impedance sensing, and microfluidic technology. Next, this article illustrated the specific analysis and applications of single-cell sequencing. Single-cell sequencing can analyze a variety of cells including CTCs, LC-DTCs, and rAAVs, and can also be used for MEMS, EIS, IMA, and to determine cell types and subtypes. Finally, this article summarized the current shortcomings and problems of single-cell sequencing, provided solutions, and tentatively gave future application directions and prospects.

The electrical manipulation and application of single cells has been studied for many years, but further developments are needed to demonstrate reliability and efficiency for the continuous separation and purification of particles with high throughput and purity. Single-cell operations and applications require continued adoption of innovations that increase sensitivity while reducing cost, scale, and complexity. How to improve the performance of current single-cell manipulation methods and simultaneously isolate and characterize single cells, and whether to provide a universal platform for medium characterization and single-cell isolation, may be a direction for future researchers to focus on. The electrical manipulation and application of single cells involves modeling, designing, and manipulating these intersecting mathematical, physical, and hardware disciplines that are relatively unfamiliar to researchers in the biological sciences [4]. In addition, many engineers have difficulty preventing cell death in the device when performing experiments because they are not exposed to basic cell biology and do not know how to properly study and maintain healthy cell lines. Therefore, if automated computers can be put into cell electrical analysis, cell death caused by various reasons can be reduced, the work of various fields can be expanded, and talents in various research fields can cooperate, then perhaps the electrical operation, analysis, and application of single cells will have broader prospects.

In the future, researchers might design microchips which could be used for battery electrical performance analysis to improve the potential of single-cell analysis, which is expected to diagnose diseases by rapidly characterizing cell electrical properties. Researchers can also explore more efficient fluorescent dyes to develop, such as quantum dots and high-tech flow cytometers, including spectroscopic and microfluidic flow cytometers [3]. In the meanwhile, with the development of materials science, a wider research horizon is slowly unfolding. For example, some researchers designed a Na/K ratio detector in single-cell scale by integrating the θ-nanopipette and functional nucleic acids, which provided research on the single-cell electrical properties of cells, membrane potential, and transmembrane ion flow with a simple approach. Moreover, benefiting from the advantage of minimal cellular damage, the nanopipette device can also be used in cell Na/K channel-related drug assessment or other relevant work [90].

In general, the electrical manipulation of single cells plays an extremely important role in driving the booming drug development business.

The electrical impedance value of a cell can reflect a variety of properties, such as cell morphology and adhesion. Changes in electrical impedance values can be studied to reveal the process of cellular signal transduction, including the effects of applied drugs. For example, CEI and EIS technologies are applied to monitor a variety of GPCRs and Gα proteins that emit different signals, which accelerates the active evaluation part of GPCR drug development and speeds up the process of developing new drugs [59].

As research progresses, more single-cell manipulation methods will be developed and applied in practice. It is hoped that this review will provide new ideas and references for scholars to make the cause of human health flourish.

## Data Availability

Not applicable.
